# ‘Time is Spine’: new evidence supports decompression within 24 h for acute spinal cord injury

**DOI:** 10.1038/s41393-021-00654-0

**Published:** 2021-07-03

**Authors:** Hari Ramakonar, Michael G. Fehlings

**Affiliations:** 1 Division of Neurosurgery, Krembil Neuroscience Centre, Toronto Western Hospital, University Health Network, Toronto, ON Canada; 2 Division of Neurosurgery, Department of Surgery, University of Toronto, Toronto, ON Canada

**Keywords:** Spinal cord diseases, Cell death in the nervous system

The timing of decompression for acute spinal cord injury (SCI) has been a topic of great debate for many years. Despite the strong biological rationale for early surgical decompression, there has, until very recently, been a paucity of high-quality evidence to support this intervention. As a result, there is substantial variability in surgical practice throughout the world with available guidelines only providing recommendations based on weak clinical evidence [1].

It is well known that acute SCI has tremendous biological, psychosocial and economic impacts on patients, families and society. In spite of advancements in our understanding of the pathophysiology of spinal cord injury and thorough investigative efforts into neuroprotection and regenerative therapies, treatment options have remained limited to targeted blood pressure control, medical therapy, external immobilization and finally, surgical decompression of the spinal cord [2].

The rationale for urgent surgical decompression includes a potential for restoring blood flow and improving perfusion whilst potentially mitigating the course of secondary injury [3]. Although there has been growing recognition that early decompressive surgery is a safe and reasonable treatment option, prior clinical studies have only been suggestive of such due to low quality data arising from small sample sizes, retrospective analyses and inconsistent methods and outcome reporting. Other recent studies have indicated that factors such as intramedullary lesion length may be more important than timing of intervention with regards to clinical outcomes in SCI [4].

To directly address this gap in clinical evidence and potentially inform future clinical practice, our group recently published our findings, which are the largest and highest quality analysis (to our knowledge) pertaining to the influence of timing of decompressive surgery for acute SCI [5]. We aimed to test the efficacy of surgery within 24 h of injury as this cut-off had been studied most frequently [6].

To achieve this goal, a pooled analysis was performed on 1548 individual patients with a SCI from 1991 to 2017. The data were derived from four high quality, prospective, multicenter acute SCI databases. These were specifically chosen as they contained the highest quality granular data, including time elapsed from injury to surgery. Included were the North American Clinical Trials Network (NACTN) SCI Registry [7], the Surgical Timing in Acute Spinal Cord Injury Study (STASCIS) [8], the Sygen trial [9] and the National Acute Spinal Cord Injury Study (NASCIS III) [10]. The mean age of the patients was 39.1 years, with 528 patients undergoing early surgical decompression within 24 h and the remaining 1020 patients undergoing later surgery.

Four major findings were apparent from this study:

1. Firstly, and potentially most importantly, surgical decompression within 24 h of acute SCI was associated with superior sensorimotor recovery at 1 year compared to patients who had their operation beyond 24 h. Patients who had early decompression surgery experienced greater improvements in mean motor scores (4.0 points [95% CI 1.7–6.3]; *p* = 0.0006), light touch scores (4.3 points [1.6–7.0]; *p* = 0.0021), and pin prick scores (4.0 points [1.5–6.6]; *p* = 0.0020). Furthermore, patients who had early surgery also had better ASIA Impairment Scale (AIS) grades 1 year after surgery.
2. In the first 24–36 h following SCI, there is a steep and continuous decline in motor recovery with delayed surgical decompression.
3. After the first 24–36 h following injury, motor recovery plateaus and the efficacy associated with early decompression is lost.
4. In cervical SCI, the additional improvement in total motor scores with early decompression is greater in the upper limbs, at or just below the level of the injury, than in the lower limbs.

The above study represents powerful evidence, which from a practical perspective indicates that ideally all acute SCI patients who are surgical candidates should undergo their procedure within the first 24 h from the time of injury. From a health care policy and quality benchmark standpoint, this should be the current target to aim for and this evidence should be used to update clinical guidelines accordingly.

From a practical and logistical standpoint however, early decompression for spinal cord injury may not be feasible in certain circumstances. For example, managing patients who are medically unstable from multiple trauma or medical co-morbidities may preclude early spinal decompression. Infrastructural capabilities such as patient transfers to tertiary spinal surgical centers, obtaining necessary diagnostic investigations and performing surgery in an ‘after hours’ environment, and (as we have all recently experienced) unforeseen circumstances, such as pandemic precautions, may provide formidable challenges in performing early decompressive surgery.

Nonetheless, the evidence is compelling and will hopefully institute a paradigm shift in clinical practice. Current data from Canada indicates that surgery within 24 h of SCI is tenable in less than 50% of patients [11]; which indicates there is much work to be done in affording better patient outcomes. It should also be noted that if a patient does not meet the 24 h threshold for decompression, this should not preclude them from urgent surgical intervention as our results do indicate a potential, albeit smaller, persistent benefit with slightly delayed intervention.

Though this study provides a robust argument for early decompressive surgery, future work is clearly required. Whilst the 24-h threshold from time of injury was chosen as the arbitrary ‘line in the sand’ defining what constitutes early surgery, there is certainly an argument to further investigate if even earlier surgical decompression would be of additional benefit. Previous smaller studies have supported so called ‘ultra early’ decompression with thresholds defined as either 8 h or 12 h following injury with promising outcomes [12, 13]. Furthermore, investigation into the heterogeneity of patients, injury patterns and surgical techniques are all areas which could help further direct clinical practice. In particular, it would be prudent to investigate those patients that are often deemed ‘futile’ due to a complete (or AIS grade A type) SCI and hence frequently do not undergo surgical decompression with the clinical urgency that perhaps they require. In addition, further work is required to define what constitutes an adequate decompression, whether there may be a role in selected cases for an expansile duroplasty and the value of intrathecal catheter-based intraspinal pressure monitoring to allow for targeted spinal cord perfusion management [14, 15]. To enhance clinical interpretation, it would be pertinent for future studies to also focus on functional and quality of life outcomes, as most prior research has focussed primarily on neurologic outcome.

In essence, acutely spinal cord injured patients should have early surgical decompression within 24 h. There is now compelling evidence to suggest this has an important bearing on long term clinical outcomes. Clinical practice guidelines and healthcare policies should be updated accordingly to reflect this. Expeditious restoration of spinal cord perfusion is critical to minimize secondary damage and protect potentially salvageable neurological tissue within an ischemic penumbra. Analogous to recent advances and policy changes associated with ischemic stroke treatments, perhaps health-care systems need to be revised to support the prompt delivery of surgical care for SCI. This affirms the concept that ‘time’ really is ‘spine’ and these patients should be treated with the appropriate urgency that we now know they require.
